# “I Didn't Make It, but…”: Deselected Athletes' Experiences of the Talent Development Pathway

**DOI:** 10.3389/fspor.2020.00024

**Published:** 2020-03-27

**Authors:** Graham Williams, Áine MacNamara

**Affiliations:** ^1^Institute of Coaching and Performance, University of Central Lancashire, Preston, United Kingdom; ^2^Millfield School, Somerset, United Kingdom; ^3^Faculty of Science and Health, School of Health and Human Performance, Dublin City University, Dublin, Ireland

**Keywords:** talent pathway, youth sport, positive youth development, transferable skills, deselection

## Abstract

The experiences of youth athletes on specialized talent development pathways has received considerable attention in both the media and literature. Despite the low conversion of pathway entrants into senior athletes, the experiences of deselected athletes have received less attention. The purpose of this study therefore was to explore the talent pathway experiences of youth athletes who were deselected from a pathway and to consider how those experiences influenced their life post deselection. Ten participants were purposefully sampled based on their prior involvement in a formalized and selective talent programme. Individual semi-structured interviews were conducted, and data was analyzed via a thematic analysis. Participants identified constructs of the talent development environment, psychobehavioral skills for future development and personal responsibility for future development as facilitators for future success beyond the talent pathway. Specifically, environmental constructs, such as high performance support systems and expectations of professionalism positively contributed toward the acquisition of transferable psychobehavioral skills, such as a determination to be successful, a confidence to back oneself and social maturity. Youth athletes reported feeling empowered to take personal responsibility for development, through attributes such as a commitment to be the best they can be and an intrinsic motivation to succeed. These findings suggest positive experiences, built around the development of transferable skills and behaviors, can accrue from being on the talent pathway. As such, the talent pathway can be a successful foundation for both success in that sport and as a facilitator of success beyond sport.

## Introduction

The pattern of selection onto, and deselection out of, talent pathways sheds light on the volatile nature of development and success in youth sport environments. Youth athletes are being recruited at an increasingly younger age (Baker et al., 2018) into formalized and selective talent pathways, despite the well-documented low conversation rates of junior to senior performers. For example, only ~2% of youth athletes engaged in a range of talent pathways achieve international honors as a senior athlete (Ackerman, 2013; Gullich, 2014; Honer et al., 2015). Gullich (2014) found an average annual turnover rate of 24.5% in all German youth elite football academies, with a probability of not being in a national age group programme 5 years after starting at above 70%. In athletics, recent research has shown that only 9% of males and 13% of females ranked in the top 20 as UK senior athletes were ranked within the top 20 as under 13 s (Kearney and Hayes, 2018). This trend is also present across Olympic sports such as swimming, volleyball and judo where <30% of junior internationals progressed to compete internationally at senior level (Barreiros et al., 2014). In rugby union, 76% of players competing at a national level as 13 year olds did not compete nationally at under 18 (Durandt et al., 2011). Collectively, this data highlights the challenging journey that youth athletes take on the road to senior success and the low probability of achieving “elite” senior performance. Acknowledging that the majority of athletes selected onto a talent pathway do not ultimately succeed in that sport at the highest level, it is important to understand the experience of being on, and then deselected from the talent pathway from the perspective of the young athlete. It may be that the talent pathway can provide a positive, and developmentally appropriate experience for youth athletes irrespective of whether they reach elite status, or not. Of course, this is a complex picture and the literature has also highlighted how high performing youth soccer players can experience psychological distress immediately following deselection (Blakelock et al., 2016), while authors have proposed the importance of programmes of support to accommodate for the risks of mental health disorders in deselected elite student-athletes (Brand et al., 2013). The mixed evidence of the nature of the deselection experience points to the importance of exploring the experiences of deselected athletes in the years post deselection. Given the considerable resources that are invested in young athletes, it seems pertinent, not least to key stakeholders such as parents, coaches, and the athletes themselves, to ensure that the development experience is positive and beneficial in the long term for as many athletes as possible.

Reflecting the non-linear nature of the development pathway, and the range of destinations that youth athletes eventually inhabit, it is important to consider how the talent pathway prepares young athletes for long-term success and participation. Collins et al. (2012) suggest re-defining excellence in talent development terms by considering achievement across a continuum of elite sporting participation, participation for personal excellence, and participation for personal well-being. In the context of youth sport, the “three worlds continuum” (Collins et al., 2012) suggests that a common foundation of appropriate early experiences enables youth athletes to move between “worlds” as they develop (Giblin et al., 2014), ultimately enabling a lifelong engagement in sport at a level commensurate with the individual's ability and motivation. The basis of the three worlds philosophy (Collins et al., 2012) requires a deliberate and purposeful focus on the skills, abilities and competencies that support opportunities for lifelong engagement in sport, increased physical activity, and greater well-being (Gublin et al., 2014; MacNamara et al., 2015; Macnamara et al., 2016) *as well as* elite sport. This approach is built on an autonomous, supportive, yet challenging development environment that facilitates the growth of the transferable skills that benefit youth athletes across the “three worlds.”

There is considerable evidence that a range of environmental factors facilitate learning and development in youth sport (Martindale et al., 2005, 2007; Henriksen et al., 2010b, 2011). Indeed, in an applied setting, Martindale et al. (2013) found a significant difference in “quality preparation” and “understanding the athlete” when assessing what was described as “high vs. low quality” talent development environments. High quality development environments have been shown to focus on the growth of intrinsic motivation and emphasis the self-development of youth athletes (Martindale et al., 2010). More specifically, talent development environments that emphasized fundamental skill progression in the context of long term development, within a robust support network, have been shown to positively influence intrinsic goal striving (Wang et al., 2011). This developmental and supportive approach may be of importance given that striving to achieve an intrinsic goal has been related to an enhanced well-being, greater confidence, greater learning capacity, and reduced stress in youth athletes (Vansteenkiste et al., 2004; Ivarsson et al., 2015). On the contrary, a focus on the pursuit of extrinsic rewards and goals (i.e., medal count, winning, achieving professional contract status) has been associated with a reduced level of perceived competence, autonomy, and relatedness (Vansteenkiste et al., 2004). As such, the design and deployment of a high quality talent development environment is vital to support *all* youth athletes in optimizing the development of a range of positive skills and capacities to help them navigate through and beyond the talent pathway.

Psychobehavioral factors have been widely identified in the literature as the mechanism that enables young athletes to cope with the various challenges of development and negotiate the inevitable transitions that they encounter as they progress within the pathway (Bjorndal et al., 2017a), between stages of development (Bjorndal et al., 2017b), and indeed out of the pathway (Avory and Rumbold, 2016). Psychological Characteristics of Developing Excellence (PCDEs; MacNamara et al., 2010a) have been proposed as a toolbox of skills and characteristics that support the talent development journey. In a sporting context, these tools have been shown to contribute to robust and positive pre and in event behaviors, enhancing learning ability, and increased personal and sporting development capacity (MacNamara et al., 2010b). PCDEs have also been recognized as supporting the development of talent across a variety of domains such as music (Kamin et al., 2007; Jarvin, 2017) and academia (Zeidner and Matthews, 2017), highlighting their transferability across performance domains. Therefore, the appropriate development and deployment of these skills should be a substantial component of any talent development pathway. Given the rocky road of talent development (Collins and MacNamara, 2012), embedding PCDEs into the talent pathway should offer youth athletes the potential to achieve within and beyond the talent pathway. It may be that these PCDEs are the (or part of the) mechanism that enables deselected youth athletes to negotiate the deselection transition and optimize their potential in an alternative domain.

Understanding and investigating the talent pathway experiences of deselected athletes has been historically challenging. Deselection research in competitive youth sport has gone some way to capture the experiences of young athletes cut from talent pathway. Authors have recognized the shared responsibility of deselection by athletes and their parents (Neely et al., 2017), deselection decision making by sports coaches (Neely et al., 2016), and changes in athletic identity following deselection (Avery and Rumbold, 2016). More recently, authors have explored why seemingly gifted youth athletes fail to achieve their potential in transitioning to senior professional status, identifying a lack of psychological resource, appropriate challenge, and positive motivational and mastery-orientated climate as causal factors (Rothwell et al., 2018; Taylor and Collins, 2019). In reality, and given the nature of talent pathways, opportunities for youth athletes to graduate to professional sport will always be slim and only a small percentage will ever “make it.” Inevitably, the “dead bodies” of talent development (i.e., those who do not progress to elite levels of performance) will continue to be vast in proportion. Despite this, there is a paucity of literature exploring youth athletes' experiences in sport post-deselection and the extent to which early engagement in talent pathways may support individuals in life *after* youth sport. This is an important area of study given the volatile nature of development and the low probability of achieving “elite” senior success as referenced previously. The purpose of this study therefore was to explore the talent pathway experiences of youth athletes who were deselected from a pathway and to consider how those experiences influenced their life post deselection. In doing so, it is hoped this information will help form a health check for talent development stakeholders on best practice in the development of generalizable psychobehavioral skills to allow youth “to do more and be more” both inside and outside of the sporting domain post deselection from a talent pathway.

## Materials and Methods

### Philosophical Positioning

The aim of this study was to develop practically meaningful knowledge about the talent development pathway in sport and, as such, our approach was driven by a pragmatic research philosophy (Rorty, 1999; Bryant, 2009). As such, this study embraces the experiences, realities and reflections of a variety of individuals previously engaged in formalized and selective talent development pathways in sport. Therefore, our aim was not to develop generalizable truths but instead to provide practically meaningful insights about athletes' experience of the talent pathway and subsequent deselection process. As such, we were able to collect subjective evidence orientated around the lived experiences of those youth athletes who transitioned through the pathway but failed to make it as senior professionals. In order to do this, a qualitative research strategy was adopted (Denzin and Lincoln, 2008) as this enabled the generation of a useful picture of the athletes' experience and probe their experiences and perceptions in detail (Strean, 1998; Feilzer, 2010).

### Participants

Ten participants were purposefully sampled based on their prior involvement (pre-18 years of age) in a formalized and selective pathway associated with either a professional rugby or cricket club in the UK. Rugby and cricket were selected as these are established talent pathways that lead to professional sport. Participants were sampled on the basis that they had been deselected prior to signing professional terms and were identified through personal networks. Participants were males aged between 20 and 25 years (*M* age = 20.6 years, *SD* = 0.7 years). Years of engagement in a formalized and selective pathway ranged from 2 to 9 years (*M* engagement = 5 years, *SD* = 2.4 years). Typically, athletes enter formalized rugby and cricket pathways at around 14–16 years of age and therefore the timeframe of the athletes' experiences is reflective of the realities of the sports selected. At the time of interview, participants had been deselected from the pathway for between ranged from 2 to 4 years (*M* years since deselection = 2.6 years, *SD* = 0.7 years). Additional demographic and sporting information can be seen in Table 1.

**Table 1:** Participant profiles.

**Chronological age range**	**Associated sport**	**Age group at selection (Under)**	**Age group at deselection (Under)**	**Years in talent pathway**	**Years post deselection from talent pathway**	**Destination post deselection from talent pathway**
20–25	Rugby	17	18	2	3	Higher Education
20–25	Rugby	15	18	4	4	Higher Education
20–25	Cricket	10	18	9	3	Higher Education
20–25	Rugby	15	18	4	2	Higher Education
20–25	Rugby	16	18	3	2	Higher Education
20–25	Cricket	12	18	8	2	Higher Education
20–25	Rugby	14	18	5	3	Full Time Employment
20–25	Rugby	16	18	3	3	Higher Education
20–25	Cricket	11	18	8	2	Higher Education
20–25	Rugby	15	18	4	2	Full Time Employment

### Procedures

Following research ethics board approval and after receiving individual consent to participate and to be quoted within the final manuscript, participants engaged in an interview process to develop an understanding of their prior experiences during their time enrolled in their associated pathway. Each participant engaged in one 45–60 min interview. The method used to collect and analyze the content of the interviews was thematic analysis (TA). This research approach was selected as it allowed the researcher to, “…see and make sense of collective or shared meaning and experiences” (Braun and Clarke, 2012, p. 2) and identify, “…what is common to the way a topic is talked or written about and of making sense of those commonalities” (Braun and Clarke, 2012, p. 2). This approach, therefore, is appropriate for extracting the participant's personal perceptions and accounts of their time in a talent development programme. In addition, it allowed the participants to interpret how these experiences may have influenced their future experience, despite not making it to the professional ranks in their sporting domain.

Semi-structured interviews were conducted with each participant. Based on the work of Bevan (2014), the key interview questions were structured around three elements. Firstly, descriptive questions allowing the participants to contextualize and extract their fundamental pathway experiences (e.g., Tell me about the time you spent in the talent pathway? What was the best/most challenging thing about being on the pathway?). Secondly, structural questions allowing the participants to engage in critical dialogue regarding more specific content of their pathway journey (e.g., Since leaving the talent pathway, which skills/experiences that you learnt/gained in the pathway have you utilized?). Finally, questioning based on imaginative variation (Giorgi, 1985) allowing the participants to explore their prior experience with enhance or adjusted terms (e.g., If you were to repeat your pathway experience again, what things would you do/you want the club to do differently based on what you know now?). Through imaginative variation, the researcher explored both concrete experiences and reflections on a variety of possibilities related to the participant's meaning of their experiences.

### Data Analysis

Interview transcripts were analyzed predominately via an inductive approach to TA. This is a bottom up approach, focused on deriving codes and themes from what is in the data (Braun and Clarke, 2012). This approach to TA has also been acknowledged as being experiential in orientation, as well as essentialist in theory (Braun and Clarke, 2012). In this context, the researcher was able to “give voice” to the experiences and meaning of the participant's responses. Braun and Clarke's six phase approach to TA (2006) was utilized during the data analysis process. Firstly, the authors familiarized themselves with the data before generating initial codes. Following this, the initial codes were collated into potential themes based on common features and then grouped together into higher-order themes based on the highest level of abstraction. Following this the themes were subjected to review and further refinements. In the final steps, the themes were defined and named by the authors according to the essence of the data codes within each theme. The second author reviewed all the annotated and coded transcript. When a discrepancy in interpretation was found, both authors referred back to the original transcript, discussed the notes and themes and agreed on a consensus position. This system was applied to all transcripts, expanding to integrate new concepts as they appeared, thus allowing referral amongst all transcripts (Smith and Osborn, 2003). During this process, the first author utilized member reflections whereby the findings were shared with participants for reflection (Smith and McGannon, 2018). The member reflection process allowed participants the opportunity to provide additional insights and accounts in order to enhance the robustness and depth of findings. All participants were sent the final transcripts of their interview via email and offered the opportunity to reply with amendments if they felt the final transcript did not reflect their talent pathway experiences shared during the original interview. Three participants responded to provide additional information to add detail and clarity to their final transcript.

## Results

Thematic analysis of the data highlighted three higher order themes (constructs of talent development environments, psychobehavioral skills development for future success and personal responsibility for future development) and 19 lower order themes. These themes are displayed in Table 2 and presented below with exemplar quotations used to illustrate the analysis.

**Table 2:** Thematic analysis.

**Raw data**	**Lower order themes**	**Higher order themes**
Inspiring training facilities Opportunity to train like a pro Insight into the demands of pro sport	High performance support systems (90%)	Constructs of talent development environments (100%)
No slip in standards Optimizing individual potential High performance behaviors	Expectations of professionalism (100%)	
A focus on the minority Favoritism clearly apparent Not the right “fit” for the club	Elitist philosophy (100%)	
“Show me you care” Lack of coach-athlete relationship Atomistic support	Impersonal approach to development (100%)	
Make or break approach to performance Lack of pressure recognition from coaches	Pressure to perform (90%)	
A focus on winning Need to satisfy coach expectations Lifelong engagement in sport as a low priority	External focus of motivation (50%)	
Accept failure courageously Hard work as a non-negotiable Desire to prove people wrong	Determination to be successful (70%)	Psychobehavioral skills for future success (100%)
A matter of choice Ability to weigh up all the options Ambition to be the best I can be	Willingness to make sacrifices (80%)	
Taking responsibility for one's schedule Managing the balance between sport and academia Personal consideration for overall well-being Structure as a necessity	Time management skills (100%)	
Clarity in communication Open and honest interactions Consistency of expectation in communication	Effective communication (80%)	
Ability to integrate into a range of social settings Optimizing the dynamics of the team Contributing to mutualistic relationships	Social awareness and maturity (100%)	
Owning the goal setting process Knowledge of what it takes to succeed	Goal setting (90%)	
Belief in one's ability Focus on expressing myself through sport Willingness to take a risk	Confidence to back myself (70%)	
Enjoyment of playing sport Internal focus of motivation	Personal satisfaction (90%)	
Establishing the right work ethic Making a purposeful contribution to the team Owning the decision-making process	Accountability for one's development (100%)	Personal responsibility for future development (100%)
Making the most of every opportunity Investing in personal development Doing the extras High expectations of one's self	Commitment to be the best you can be (90%)	
Desire to achieve a personal goal Freedom of choice to define what success looks like	Intrinsic motivation to succeed (90%)	
Significant others who believed in me Significant others who helped me Importance of establishing a diverse support network	Support mechanisms to fulfill potential (100%)	
Learning takes time and effort “Shoulda, woulda, coulda” Critical self-analysis of areas for improvement	Self-reflection (100%)	

### Constructs of Talent Development Environments

All the participants reported how a variety of talent development constructs influenced their development during their time in the talent pathway. Exposure to high performance support systems was identified by nine participants as being both a positive and enabling part of their development. Participants referenced the opportunity to train like a professional sportsperson and the inspirational nature of the training facilities, both structurally and motivationally, that they were exposed to. For example, participant 10 identified that they enjoyed:

…*getting involved in the professional environment, seeing the standards which the pros have. Getting put in that environment was good for my head, good for the way I thought about myself, my rugby, my skills as a number nine. It was massive*.

In addition, the talent development environment was noted as providing an insight into the demands of professional sport by four of the participants. Typifying this, participant 7 commented:

*We would run through a professional rugby player's day. Train on field, do a gym session, have lunch, receive nutritional information. It was all very professional*.

Indeed, previous research has explored the transitional “culture gap” between senior professional and junior academy level sport (Relvas et al., 2010), with authors proposing that early exposure, normalizing operational mechanisms, and the ability to adjust to explicit practices as vital to navigating this “gap” (Morris et al., 2014; Roynesdal et al., 2018). The value and transferability of these formative experiences in a talent pathway to experiences in other areas is supported by literature from the business and academic domains in which reciprocal engagement, effective work inductions and the regulation of professional practice has enhanced transition success (Pentland, 2012; Shockley et al., 2013). Indeed, participant 1 stated:

*Training with the pros at their training base, it was the sense that you were almost professional. The coaches were in the kit, you were using the same facilities as the pros. It was a lot different to just playing for school. It was all such a high standard. It was knowing what the standard was like and where you want to be. When I went to university (post deselection), my aim always to push on and get to the highest level in whatever I was doing. The academy programme gave me more experience in doing that and what it takes. That professional attitude put me in the mind set of, right, do your work, revise hard and you do it because at the end there will be benefits*.

It may be therefore, that the ability of youth athletes to experience and normalize the context of a professional domain during their time in a pathway, can genuinely support the long term success of those who “make it” in sport *and* those who do not.

Exposure to high performance environments also exposed participants to the expectations of professionalism that was demanded of them by the sporting organization. Seven participants referenced how the environment drove high performance behaviors, such as “no slip in” standards and an individualized approach to optimizing potential as key mechanisms of progression. As a result, participants noted how this emphasis on displaying and maintaining high professional standards had a positive impact on them post-deselection:

*From being in that environment, I think the difference between good players and really good players is the best guys get on with it and know what they need to do to improve*.

*Their standards are so high. They take a lot of ownership of their game and improving their skills. I have definitely taken that away with me and applied it to other aspects of my life, such as my university studies*. [Participant 6]

As referenced here, the impact of high performance support systems at a macro level and expectations of professional behaviors at a micro level was portrayed to have a positive transfer both during the participants' time on the talent pathway and following their deselection. These findings mirror the work of Henriksen et al. (2010a) in which macro and micro-environmental factors were proposed to impact the present and future capabilities of prospective athletes in both athletic and non-athletic domains. In addition, authors have recognized the need to create a positive motivational climate for youth athletes to reduce the risk of dropout and de-motivation in formalized and selective talent pathways (Rothwell et al., 2018). Finally, evidence suggests progress from youth to senior athlete may be hindered if youth athlete fails to display the expected high performance behaviors and attitudes, as defined in the senior professional environment (Roynesdal et al., 2018). The findings from this study provide evidence to suggest how constructs of talent development environments, such as underlining values, beliefs, and standards, have the potential to positively impact the personal development of those youth athletes who fail to progress to senior professional sport.

Contrary to these facilitative environmental constructs, participant also identified experiences that may have hindered future development. All participants referenced an elitist approach to programme delivery in which favoritism was apparent from the coaching staff toward the perceived “best players” in the squad. For example, participant 6 stated:

*I felt like there was quite a big divide between the programme I was getting and the opportunities I had versus some of the other players in programme. From my perspective, I felt we were all in the same boat, but at training sessions for example, coaches would spend more time with certain players. They would get filmed by the coaches and have a chance to talk about the footage. I felt most of the time I was just a net bowler*.

In addition, five participants acknowledged that they were not the “right fit” for the club that they were signed to:

*It was obvious I was at the end of the journey with the club. At that point my Dad and I left the meeting. That was it for me. I was angry; I was putting in so much effort on and off the pitch. I thought I was good enough, I had played at the top age group level, but just because the coach did not take a liking to me, I did not get offered a deal. There were obviously some very good coaches, guys who had played at the highest level. It would have been great to get more from them. It felt like they were trying to change us to suit the way they wanted to play rather than improve us to give us the best chance of making it*. [Participant 10]

Reflecting this, all of participants expressed how they felt that an impersonal approach within their pathway experience negatively impacted on their progression, both from a playing perspective and on their motivation and confidence in the sport. For example, six participants described the need for coaches to “show me you care” especially during difficult “release or retain” transitions on the pathway. In addition, seven of participants referenced a lack of positive coach-athlete relationship with their pathway coaches and how this impacted on their experience on the pathway. This finding is notable as Jowett and Shanmugam (2016) state, “…it takes two to bring about change such as skill development and performance success. Neither the coach nor the athlete can do it alone!” (p. 18). The 3+1Cs model for optimizing coach-athlete relationships can be utilized as a reference point for appraising the perceived effectiveness of such relationships. The findings of this study suggest participants experienced a potential paucity of closeness, commitment and co-orientation:

*It would have been better if they wanted to get to know you and it would have been a better experience. It would have given you a better outlook. If people asked what it was like being in the academy, some might say it was good, some would not because they didn't get to know us that well. I would like to think that if I was in the coach's position, I would have wanted to get to know people, get them to where they wanted to be, but also be more personable about it*. [Participant 1]

*We did not get into those deep coaching conversations. The sessions were very open, week by week. We did not have clarity on what we needed to work on because there were so many players at training. I found it hard to feel like I was improving at the club*. [Participant 4]

The discord between optimal coach-athlete relationships and those experienced by the participants in this study is concerning given the plethora of literature endorsing the added value of productive dyadic relationships (Reeve, 2012; Rogat et al., 2014; Jowett and Shanmugam, 2016). These authors identify that coaching is more than directing an athlete to perform a task. The coach, and a positive coach-athlete relationship, can have beneficial impacts outside the sporting domain, enhancing perceptions of self-competence and overall worth. The risk of impersonal coach-athlete relationships for those who “won't make it,” as referenced above, is that these youth athletes, deselected from the talent pathway, may experience impaired perceptions of personal growth and development.

The participants' descriptions of an “impersonal” talent development experience may relate closely to the motives of the sporting organization and their stakeholders. More specifically, participants identified a narrow focus on them as a sports person and a large emphasis placed on their performance on the field of play as described below:

*Obviously, the club are there to make you a professional rugby player. You do not go there to make friends. You do make friends, but you are there because you are good enough, they put time into you because they think you are good enough and they want to make you better at rugby. They would not be that interested in you as a person, I would not have thought. I can kind of see why they do not get personable. It is a business, I guess*. [Participant 1]

The impact of this approach, and it's influence on identity formation, was felt by the participants in their transitions post deselection:

*Providing opportunities and sharing experiences outside of the club would have been so beneficial for me and the other players. I went on to play at national club level, but the academy didn't help with any of that. I know a lot of players who had the same phone call as me, stopped playing rugby and haven't picked up a ball since. We got a lot from being incredibly committed rugby players and suddenly there wasn't a second option. I can't see how that is fair*. [Participant 7]

The ability of pathway stakeholders to facilitate reflection on the added value of being there or thereabouts on a talent pathway for deselected youth athletes may be critical to their success in life after youth sport. In addition, participants felt that the coaching staff failed to respond to the stressors that involvement in a talent pathway placed on the youth athlete. Most notably, participants acknowledged that these stressors may have contributed to the drop out from sport once deselected from the talent pathway, for example participant 7 stated:

*At the club there was pressure from coaches. I did not feel like it was intentional, and I did not mind it, the mentality that is. It was like a make or break situation. I do not think they appreciated the mental and physical demands on young guys in the academy programme. As I said before, there were lots of players who stopped playing rugby when they heard they were being released*.

Evidence has suggested that talented youth athletes that experience premature termination in sports participation may be at greater risk of physical and mental well-being issues (Brown and Potrac, 2009; Crane and Temple, 2014). This increased risk is concerning given the narrative reported above and collectively highlights the responsibility that talent development stakeholders have in ensuring positive outcomes for *all* youth athletes engaged in formalized and selective talent pathway. Such findings corroborate with the work of a range authors in the field of talent development who have highlighted the prevalence of performance outcomes within youth sports programmes such as a “win at all costs” mentality and rigid and inflexible development pathways (Collins and MacNamara, 2012; Rothwell et al., 2017). The evidence in this study suggests constructs of the talent development environment can create experiences and a climate that both enable and challenge the development of the youth athlete through the talent pathway. Talent pathway stakeholder's must be cognizant of the implications of the talent development environment on the inter and intrapersonal development of those that are deselected prior to signing professional terms, as when positioned correctly, these constructs can support the development of transferable skills through and beyond sport.

### Psychobehavioral Skills for Future Success

All participants highlighted the positive contribution that the development of a range of psychobehavioral skills played on their success post talent pathway. This finding is consistent with previous literature exploring the role of learning strategies to support the development process (Cropley et al., 2017; Jarvin, 2017). Within this study, seven participants identified their determination to be successful as an important quality that fostered and supported future achievement. For example, participant 6 described how this determination was nurtured during this pathway experience:

*I think I like proving people wrong. For example, when I got dropped from the academy programme the first time around as a batsman, I remember thinking, right let's become a bowler and get back into the programme. It was the same for my GCSEs. People said that I should not have done certain A levels as well and I was not smart enough because of my GCSE grades. I kind of needed that to prove them wrong, it made me work harder to show them and myself what I could achieve*.

This approach was supported by the participant's willingness to make sacrifices and a confidence “to back themselves.” These psychobehavioral skills align well to the constructs of positive youth development, defined as the “…development of personal skills or assets, including cognitive, social, emotional, and intellectual qualities necessary for youth to become successfully functioning members of society” (Weiss and Wiese-Bjornstal, 2009, p. 1). Expanding further, this study found that nine of the participants referenced the development of personal assets such as: time management, effective communication, social awareness, and goal setting. The participants described the systematic development of behavioral and social skills that were reinforced through early talent pathway experiences and deployed outside of the sporting domain. This approach presents a new insight into the added value of “being there or thereabouts” in a formalized and selective sports programme.

As referenced previously, the value of psychobehavioral skill development in sport is well-understood. Literature has highlighted the importance of this skill development process in optimizing individual development capacity via practice, learning, and overcoming adversity in sporting competitions (Orlick, 1996; Gould et al., 2002; MacNamara et al., 2010b; Collins et al., 2016). However, the context of this study provides encouraging insights and potential reassurances that the process of talent development in youth through sport may also provide transferable and highly valuable personal assets that can be deploy in alternative domains post deselection. For example, participant 1 stated:

*The goal setting process was useful. I didn't write anything down, but I knew if I was going to start for the first team I had to be at the right physical level and continue to improve my skills. On the academic side, now being injured, my aim is to push for a first. I know I need to spend an hour in the library, crack on in revision periods and put the work in so I know what I am doing and give myself a chance to get good grades. I think the academy experience opened my eyes up to this area, the stuff like time management and taking ownership, doing stuff for yourself and setting goals*.

Supporting this, participant 8 stated:

*Awareness of others was a really big thing for me. I try to make good impressions on people, and it becomes easier to read other people. You can figure people out quite quickly when you have been involved in so many teams through a rugby academy programme. It's actually a really great trait for me. I think it will help me in business. It is going to help me get an idea of how I can be a good team player and take my time to make sure I make a good impression*.

As referenced in the quotes above, the common foundation of early experiences and subsequently learnt psychobehavioral skills may empower youth to achieve across a wider continuum of outcomes. More specifically, the development of psychobehavioral skills through the talent pathway can contribute to success post deselection from youth sport and is transferable to domains such as academia and business. This message is supported by the work of Taylor and Collins (2019) who identified a paucity of psychological resource and challenge as notable factors contributing toward the derailment of talented youth athletes. The findings in this study would suggest that the systematic and timely promotion of psychological challenge and support can enhance the psychobehavioral skills of those youth athletes that are there or thereabouts on a talent pathway. Moreover, these findings suggest that the development of such skills are transferable and beneficial for youth athletes post deselection from the talent pathway. Critical to this transfer may be a supportive network of stakeholders as identified below:

*There were lots of great coaches that I enjoyed working with and my parents were very supportive. However, I would say one of the other players, he is a good mate and his parents did a lot of the driving and helping out like that. They knew all the coaches really well, so they would help me if I was struggling and they offered some great advice on how to communicate best with the coaches. In terms of coaches, it was strangely the forwards coach who I felt connected with me. He gave honest feedback, set me extra tasks for my skills and would tell me to get my head up if I was struggling. He kept me motivated and was really genuine. Being part of the academy programme has made me realize that every bit of work that you do can contribute to your improvement*. [Participant 5]

Participants in this study recognized that supportive others who challenged and supported them in the development of psychobehavioral skills provided opportunities for them to thrive at that time *and* in life post deselection.

### Personal Responsibility for Future Development

All participants referenced the importance of taking personal responsibility for future development as an attitude acquired from their pathway experiences. Participants were able to provide examples of how the work ethic they developed in the pathway set them aside from their peers in future performance domains:

*The other one is self-motivation. I think I have a real drive and desire. I am prepared to get out of bed and do the hard work. At university there is no consequence for missing training. We have a big training squad, but I still want to improve and play the best I can. The guys from academy backgrounds are always on time, always push to get better and adapt quickly to the demands expected of them*. [Participant 9]

*It is about making the most of the opportunity. There are lots of guys in the programme at the moment who are more talented than me, but they do not turn up to training or go to the gym. They are so lazy. They have not had the programme or experiences behind them, learning from mistakes and lessons and using that to get better*. [Participant 3]

Such findings correlate with the work of MacNamara et al. (2010a) who identified the importance of a multiplicative understanding of development. In this context, participants were able to recognize character strengths gained from prior developmental experiences, such as their ability to invest in their personal development and contextualize how these strengths supported achievement in their current domain. Participants were able to reference their formative pathway experiences as positively enhancing such characteristics, giving credence to talent pathways as rich and positive learning environments for all youth athletes. Recognition of such competence and confidence by the participants in this study aligns well to components of Lerner's 5C's of positive youth development (Lerner et al., 2005). The 5Cs model of positive youth development has conceptual overlap across social, academic, vocational and sporting domains. In this light, the ability of the participant's pathway experience to enhance positive self-worth (confidence) and self-image (competence) post deselection from a talent pathway and in an alternative domain, may be a critical and profound finding of this study highlighting the transferable nature of positive youth development to other domains.

In addition to the character strengths, nine participants recognized the development of an intrinsic motivation to succeed via their pathway experiences and in life beyond youth sport:

*For me it came down to how much I wanted it. I wanted to improve, I wanted to play as well as I could. It was not about doing things in half measures. At the end of the day, it was down to you and down to how much you wanted it. It was amazing to represent the club and play for the badge, but personally it meant a lot for me to show what I could do at that level. Ultimately, I did not get offered a contract, but nobody can take those memories away from me and I learnt so much along the way. That desire to improve and perform to the best of my ability is still with me now*. [Participant 8]

This individual participant's data aligns well to the macro theory of self-determination (Deci and Ryan, 2000). More specifically, participants referenced the development of autonomous motivation, in which participants developed the desire to achieve personal goals and freedom of choice to define what success looks like. This is an important finding since previous research has highlighted an individual's readiness to perform a given behavior is closely associated to their level of autonomous motivation (Sarrazin et al., 2002; Alvarez et al., 2012; Keshtidar and Behzadnia, 2017). The development of autonomous motivation shows how facilitative constructs of development environments, such as the ones in this study, and the development of psychobehavioral skills, may support young people to believe and achieve beyond a talent pathway in sport. In this context, talent pathway stakeholders may be empowered to recognize the pathway experience as a holistic and positively enabling development environment that can support personal development for and beyond the domain of sport.

In line with this, all participants acknowledged the critical role that significant others played and continue to play in their development. Participants referenced a variety of individuals and approaches that allowed this support to contribute to increasing opportunities to fulfill their potential through and beyond sport:

*One of my friends helped me loads and I learnt a lot from him. I felt like he knew me better than I did, which is very strange. He would help clarify my own thoughts, would be a good listener and sometimes help frame stuff for me. He got what I said because he had been through a similar process*. [Participant 6]

*He (assistant coach) was constantly talking to me, asking me what I did well, why I did it, what I could do better. You get a deeper thought process about it when you're playing and that helps your game. It helps you make better decisions. He seemed genuinely interested in player's development, rather than just winning. It is why I related to him well*. [Participant 4]

Importantly and as referenced here, participants acknowledged how significant others who believed in them (*n* = 5) and who helped them (*n* = 9) supported their development during and after their pathway experiences. Such findings relate well to previous literature on the benefits of parental inputs and behaviors matching their child's motives and the importance of shared goals to synchronize supportive behavior (Elliot and Drummond, 2017; Strandbu et al., 2017). This is concept is highlighted below through this study:

*Keeping the high standards and high expectations was tough, but I think it was good for me. My parents expected me to manage my school work and rugby to the highest level possible. That was a given. That was good for me. Now I know I can achieve things and set expectations of myself where I can be successful. If people doubt me or question my ability, I feel like I can stick to my standards and expectations and deliver something which is good. I enjoyed it, as well, when the coaches at the academy and England pushed me to get better. I knew that they wouldn't do that if they didn't care and didn't think I was able*. [Participant 10]

Advancing these concepts further, the findings from this study highlight that supportive and adaptive behaviors from a diverse network of friends, family and coaches may empower youth to seek to fulfill their potential by helping to frame reflective practice and refine opportunities to enhance learning experiences. This finding is akin to other researchers who have found a mixed methodology of context and interactions to provide a more rounded developmental approach, contributing to broader psychobehavioral enrichment (Japp, 2011; Hafen et al., 2012; Jang et al., 2012; Reeve, 2012). This is critical in the context of talent development given the majority of youth athletes will fail to make the transition to senior professional sport. Thus, the acquisition of psychobehavioral skills and the motivation to take personal responsibility for development in life beyond the pathway seem critical markers of success for all youth athletes at any stage or age of the talent pathway.

## Discussion

Deselection from development pathways is an inevitability for all but the most talented athletes. Although the participants in this study were, at least at that moment in their career, disappointed about deselection, they all were able to reflect on how their experiences on the talent pathway provided them with a rich learning experience that benefited them beyond youth sport. The participants noted how their early experiences in a formalized talent pathway enabled them to develop a tool box of psychobehavioral skills that supported their transitions to new environments following deselection. On face value, talent pathways may appear to be specialized environments focused on producing the next star in that particular sport. On further inspection, and as evidenced in these findings, talent pathways that focus on the deliberate preparation of youth athletes can concurrently support the development of the next superstar *and* the development of the transferable skills that enable the majority of athletes to achieve elsewhere at a level commensurate with their ability, interest, and/or motivation. Indeed, the participants in this study describe how formatively sound experiences of talent development pathways prepared them for life beyond that sport. More specifically, participants recognized the added value of professional expectations placed upon them for achievement inside and outside the sporting domain. Exposure to, and experience of, professional sport training facilities and systems of practice were seen to be inspirational and insightful, providing a head start for the participants to transition into an alternative performance domain and therefore provided opportunities to “be more and do more” beyond sport.

The participants in this study were sensitive to the impersonal, elitist approach employed by some coaches within the talent pathway, acknowledging that certain individuals were given preferential treatment based on their likelihood of achieving professionalism in sport. An impersonal and elitist approach was reflected in participants describing maladaptive coach-athlete relationships, with a focus on extrinsic motivational factors. Such findings highlight the need for talent pathways to be capable of accommodating multiple worlds of participation in which the teaching and learning of skills can support the development of a range of transferable psychobehavioral skills for all youth athletes. Such capability may be particularly important to recognize and pay tribute to in and around the process of deselection from talent pathways. For example, previous research has highlighted the potential of deselected youth athletes to be susceptible to experiencing psychological distress that may reach clinical levels (Blakelock et al., 2016). The development of a range of psychobehavioral skills and attitudes, such as determination to be successful, confidence to back oneself and social awareness was referenced as enabling participants to believe in themselves and achieve success in performance domains such as academia and business. As such, the deselection process should be a time whereby talent pathway practitioners facilitate an awareness of potential intra- and interpersonal skills developed by the youth athlete and the potential for these skills to be utilized in alterative domains. In this light, participants identified how the talent pathway encouraged them to take personal responsibility for their future development by recognizing the presence of, but not limited to, intrinsic motivation and a network of significant others to facilitate success beyond sport. Equally as important may be the lag time between deselection and the ability of youth athletes to make sense of their pathway experiences. Evidence from this study suggests the ability to engage in reflection, supported by the cultural resources available, helped the participants to make sense of their experiences and find positive meaning from the negativity of deselection. We are currently exploring, through a cross-cultural longitudinal study, the role of reflection and social support as key mechanisms of successful development in sport and between domains (e.g., education).

Taken globally, these finding are consistent with the success markers associated with positive youth development. For example, research in this area has referenced the impact of meaningful opportunity interventions and the empowerment of youth to be accountable for their own development (Eichas et al., 2017). Eichas et al. found positive youth development interventions to have cascades and “spill over” effects on several different but related inter and intrapersonal qualities in youth. Whilst the challenging and contingent relationship between sport and youth development has been well-researched (Holt et al., 2008; Weiss, 2008; Weiss and Wiese-Bjornstal, 2009; Coakley, 2011), the findings of this study highlights the potential positive and transferable developmental outcomes for youth athletes previously engaged in formalized and selective sport development pathways. Despite the participants of this study being deselected prior to signing professional terms, they describe psychobehavioral skills and effective performance strategies akin to those reported by elite athletes (Collins et al., 2016; Burns et al., 2019). Therefore, the findings of this study can be seen to support the connotation that the intentional and skillfully developed early experiences of youth athletes within a talent pathway can act as a rich development opportunity to acquire and deploy a range of psychobehavioral skills both in and beyond sport. This finding goes further to support a holistic *and deliberate* developmental approach in which talent pathways in sport should have the potential to support multiple worlds of participation and multiple outcomes of success within the same development programme. In this context, talent development stakeholders should recognize the need for the deliberate preparation of skills, abilities and competencies delivered through the vehicle of sport for youth athletes at every ability level.

The findings of this study notwithstanding, there are limitations that should be brought to the reader's attention. Given the retrospective nature of enquiry, there is a risk of recall bias. Given that participants were on average 2.5 years post deselection from a talent pathway, recall bias and thus information bias may be a threat to internal validity and credibility. However, while participant's views may have some degree of error associated to them, the experiences and skills they attained during their talent pathway journey were identified as positively enabling their success in an alternative domain post deselection. Indeed, it may be that the delayed time period post deselection from the talent pathway was required to allow the participants to truly reflect on the benefits of the pathway despite not achieving professional sport. It is important to reference the diversity within the group of participants. Whilst three participants spent 8 to 9 years in a talent pathway, three others spent only 2–3 years in a talent pathway. Future research should unpack the influence of the timing and duration of youth athlete's enrollment on talent pathways and the influence of this on their journey through and beyond the pathway. In addition, the findings reflect the experiences of participants through talent pathways in two sports: rugby and cricket. It may be that sports with more or less intensive talent pathways generate environmental constructs and intra and interpersonal development opportunities that are more or less impactful on the future success of those deselected prior to signing professional terms. Future research in this area may therefore consider the true value of the talent pathway across a broader range of sports programmes. Exploring how talent pathway coaches manipulate the development environment and facilitate reflection on lived experiences with youth athletes may go some way to capturing the in-action learning associated to personal development for future success prior to deselection. In this light, the longitudinal investigation of the lived experiences of youth athletes in a talent pathway and the associated challenges would assist in providing more valid and reliable profile of facilitators of future success post deselection. Finally, participants were recruited for the study via personal networks. Knowledge of the participants and the participants' knowledge of the interviewer may have resulted in participants refraining from disclosing certain topics or issues.

The results of this study may help guide a deliberate preparation curriculum for practitioners in the field of talent development to add value to youth athletes who are “there or thereabouts” in formalized and selective talent pathways. The professionalization of talent pathways is unlikely to change in the near future and the race to produce the next sporting superstar is likely to intensify. Talent pathway stakeholders at all levels will soon be under pressure to rationalize and evidence a more holistic and inclusive developmental approach (Mathorne et al., 2020). The evidence in this study sheds new light on the added value of talent pathways in sport as rich developmental learning environments that can support and enhance success beyond professional sport via spillover effects into alternative domains. In doing so, this study can provide a “health check” for talent pathways, ensuring the delivery of a rich learning experience for *all* young athletes is optimized and the positive and developmental cascades of learning for individuals entering any performance domain are recognized. As such, it may be proposed that the best foundation for tangible, lifelong development within and through talent pathways should focus on optimizing the potential of experiences, drawn from the environment, to support personal motivation and psychobehavioral skills for every individual at any stage of participation in a talent pathway.

## Data Availability Statement

The datasets generated for this study are available on request to the corresponding author.

## Ethics Statement

The study, involving human participants was reviewed and approved by the Ethics and Integrity Unit within Research Services at the University of Central Lancashire. The participants provided their written informed consent to participate in the study.

## Author Contributions

GW and ÁM contributed to the conception and design of the study. GW performed the data collection and initial thematic analysis, with ÁM contributing to reviewing, naming, and producing the final report, and wrote the first draft of the manuscript. ÁM reviewed and wrote sections of the manuscript. All authors contributed to manuscript revision, read, and approved the submitted version.

### Conflict of Interest

The authors declare that the research was conducted in the absence of any commercial or financial relationships that could be construed as a potential conflict of interest.
